# Map-Based Cloning and Characterization of a Major QTL Gene, *FfR1*, Which Confers Resistance to Rice Bakanae Disease

**DOI:** 10.3390/ijms25116214

**Published:** 2024-06-05

**Authors:** Hyeonso Ji, Kyeong-Seong Cheon, Yunji Shin, Chaewon Lee, Seungmin Son, Hyoja Oh, Dong-Kyung Yoon, Seoyeon Lee, Mihyun Cho, Soojin Jun, Gang-Seob Lee, Jeongho Baek, Song Lim Kim, Il-Pyung Ahn, Jae-Hyeon Oh, Hye-Jin Yoon, Young-Soon Cha, Kyung-Hwan Kim

**Affiliations:** 1 Department of Agricultural Biotechnology, National Institute of Agricultural Sciences, Rural Development Administration (RDA), Jeonju 54874, Republic of Korea; yunji.shin8@gmail.com (Y.S.); linewind@korea.kr (S.S.); hja-oh@hanmail.net (H.O.); dkyoon11@korea.kr (D.-K.Y.); hklee0214@korea.kr (S.L.); chomi1@korea.kr (M.C.); jsoojin98@korea.kr (S.J.); kangslee0227@gmail.com (G.-S.L.); firstleon@korea.kr (J.B.); greenksl@korea.kr (S.L.K.); jinhyung@korea.kr (I.-P.A.); jhoh8288@korea.kr (J.-H.O.); hyejinyoon@korea.kr (H.-J.Y.); yscha63@korea.kr (Y.-S.C.); biopiakim@korea.kr (K.-H.K.); 2 Department of Forest Bioresources, National Institute of Forest Science, Suwon 16631, Republic of Korea; kscheon16@korea.kr; 3 Department of Central Area Crop Science, National Institute of Crop Science, Rural Development Administration (RDA), Suwon 16429, Republic of Korea; wowlek44@korea.kr; 4 Department of Southern Area Crop Science, National Institute of Crop Science, Rural Development Administration (RDA), Miryang 50424, Republic of Korea; 5 Coastal Agriculture Research Institute, Kyungpook National University, Daegu 41566, Republic of Korea

**Keywords:** rice, bakanae disease, resistance, LRR-RLP, selection marker

## Abstract

Bakanae disease (BD), caused by the fungal pathogen *Fusarium fujikuroi*, is a serious threat to rice production worldwide. Breeding elite rice varieties resistant to BD requires the identification of resistance genes. Previously, we discovered a resistant quantitative trait locus (QTL), *qFfR1*, in a Korean *japonica* rice variety, Nampyeong. In this study, we fine-mapped *qFfR1* with a Junam^*4^/Nampyeong BC_3_F_3_ population and delimited its location to a 37.1 kb region on chromosome 1. Complementation experiments with seven candidate genes in this region revealed that *OsI_02728* is the gene for *qFfR1*. This gene encodes a protein with a typical leucine-rich repeat (LRR) receptor-like protein structure. RNA-sequencing-based transcriptomic analysis revealed that FfR1 induces the transcription of defense genes, including lignin and terpenoid biosynthesis genes, pathogenesis-related genes, and thionin genes. These results may facilitate investigations into the molecular mechanisms underlying BD resistance, including molecular patterns of *Fusarium fujikuroi* interacting with FfR1 and players working in signal transduction pathways downstream of FfR1, and the breeding of new BD-resistant varieties by providing a BD resistance gene with its precise selection marker. This will contribute to efficient control of BD, which is becoming more prevalent according to temperature rises due to climate change.

## 1. Introduction

Bakanae disease (BD), also called “foot rot disease”, is an important disease of rice (*Oryza sativa*), causing yield losses of 3.0–95.4% [1], and has been reported in almost all rice-growing regions worldwide [1,2,3,4,5,6,7,8,9,10]. BD is caused by the fungal pathogen *Fusarium fujikuroi*, which can thrive in temperatures as high as 35 °C [1], allowing this disease to reproduce more rapidly and spread as temperatures increase due to climate change. The infected plants show elongated shoots, yellowing leaves, stunting, seedling rot, and barren ears [11]. Conidia formed at the tillering stage are dispersed by rain splashes or the wind during the flowering stage and infect the floral organs, infecting the seeds at later stages [11]. These infected seeds are the major source of disease transmission, although the pathogen can also overwinter in dry soil [11]. The main method of controlling BD is disinfection of the seeds by chemical fungicides [1,11]; however, fungicide-resistant strains have emerged, making its control difficult [12,13,14,15].

Planting resistant varieties is an effective, economical, and environmentally friendly alternative disease-control strategy [16]. Several large-scale screens have identified resistant germplasm [17,18,19,20,21], and QTL-mapping studies using biparental mapping populations have identified 14 quantitative trait loci (QTLs) (*qB1*, *qB10*, *qBK1*, *qBK1.1*, *qBK1.2*, *qBK1.3*, *qBK3.1*, *qFfR1*, *qBK1^WD^*, *qBK1^(Z)^*, *qBK2.1*, *qBK4^T^*, *qFfR6*, and *qFfR9*) [16,22,23,24,25,26,27,28,29]. In addition, 16 QTLs (*qBK1_628091*, *qBK4_31750955*, *qBK1.4*, *qBK1.5*, *qBK1.6*, *qBK1.7*, *qBK3.2*, *qBK4.1*, *qBK6.1*, *qBK6.2*, *qBK6.3*, *qBK8.1*, *qBK10.1*, *qBK10.2*, *qBK10.3*, and *qBK11.1*) were detected through genome-wide association studies [17,30]; however, none of the genes underlying these QTLs has been identified.

Molecular studies have revealed some clues to potential resistance genes. The rice WRKY transcription factor gene *OsWRKY114* and the rice glutaredoxin gene *GRXS15* confer resistance to *Fusarium fujikuroi* when overexpressed [31,32]. A transcriptomic analysis comparing the resistant rice cultivar Selenio and the susceptible cultivar Dorella infected with *Fusarium fujikuroi* showed that the genes encoding PATHOGENESIS-RELATED 1 (PR1), germin-like proteins, glycoside hydrolases, MAP kinases, and WRKY transcription factors were upregulated in the resistant cultivar [33]. Another transcriptomic analysis comparing the resistant cultivar C101A51 and the susceptible cultivar Rasi showed that the genes encoding cysteine proteinase inhibitor 10, disease resistance protein TAO1-like, oleosin 16 kDa-like, PR1, PR4, BTB/POZ, MATH domain-containing protein 5-like, and alpha-amylase isozyme were upregulated in the resistant plants [34].

In a previous study, we found a major rice QTL for BD resistance, *qFfR1*, in the 22.56–24.10 Mb region of rice chromosome 1 by mapping QTLs with an F_2_:F_3_ population derived from a cross between a resistant variety (Nampyeong) and a susceptible line (DongjinAD) [35]. This QTL was found again in another study using an F_2_:F_3_ population derived from a cross between the same resistant variety (Nampyeong) and a different susceptible variety (Junam) in a similar position of 21.36–24.37 Mb on rice chromosome 1 [22]. In this study, map-based cloning of this QTL revealed that a leucine-rich repeat (LRR) family gene, *OsI_02728*, is the gene underlying *qFfR1*. We also developed a molecular marker for this gene, providing an important resource for the development of varieties that are resistant to BD. Our results thus enhance our understanding of the molecular basis of BD resistance in rice.

## 2. Results

### 2.1. Fine-Mapping and Identification of qFfR1

**Figure 1:**
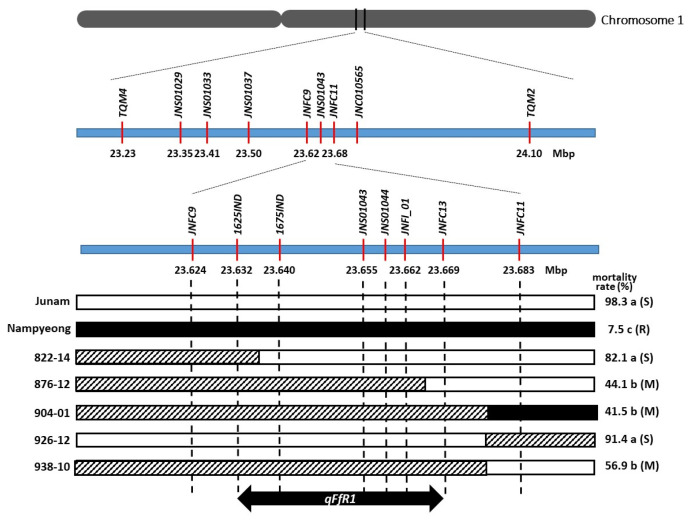
Fine mapping of *qFfR1*. Open bars represent the homozygous Junam (susceptible) genotype; black and hatched bars represent the homozygous Nampyeong (resistant) and heterozygous genotypes, respectively. Different lowercase letters on the right-hand side of the mortality rates indicate significant statistical differences at *p* < 0.05. S, M, and R indicate susceptible, intermediate, and resistant phenotypes, respectively.

We performed the fine-mapping of *qFfR1* with a Junam^*4^/Nampyeong BC_3_F_3_ population comprising 2995 plants. In the *qFfR1* mapping study, the LOD peak was located in the 23.41–23.63 Mb interval [35]. Taking this as the target region, we developed two markers flanking this region, *TQM4* (23.23 Mb) and *TQM2* (24.1 Mb), and selected recombinants in the target region by genotyping the plants using these two markers. In total, 153 recombinant lines were selected and were further genotyped using 12 markers within this region (Figure 1). The mortality rates after infection with the pathogen were measured using seeds harvested from each line. By analyzing the co-segregation of the marker’s genotype and phenotype (Figure 1), the location of *qFfR1* was mapped to the 37.1 kb region delimited by the markers *1625IND* and *JNFC13* (23.63–23.67 Mb).

This region contains three LRR family genes similar to *Cf-2*, a tomato (*Solanum lycopersicum*) gene that confers resistance to leaf mold disease caused by the fungal pathogen *Cladosporium fulvum*. First, two LRR family genes (*Os01g0601625* and *Os01g0601675*) were selected for testing as candidate genes for *qFfR1.* We performed complementation experiments by cloning the genomic regions of these genes from Nampyeong and introducing them into Junam by transformation. The resulting transgenic lines did not show increased resistance (Figure S1); therefore, these candidates were not the *qFfR1* gene.

Through this experiment, we found that the genomic sequences of these genes in Nampyeong were identical to those of the *indica* reference genome sequence (93-11) but quite different from the *japonica* reference genome sequence (Nipponbare) (Figures S2 and S3). It was therefore inferred that the Nampyeong variety contains part of the *indica* genome in the *qFfR1* region. We detected single-nucleotide polymorphisms (SNPs) on chromosome 1 in Junam and Nampyeong in comparison with the *japonica* (Nipponbare) and *indica* (93-11) reference genome sequences using resequencing data produced in previous studies [35,36]. In comparison with Nipponbare, Nampyeong had many more SNPs than Junam in the *qFfR1* region, while it had far fewer SNPs than Junam in the *qFfR1* region when compared with 93-11 (Figure S4). This supported the inference that part of the *indica* genome was introgressed into Nampyeong in the *qFfR1* region.

**Figure 2:**
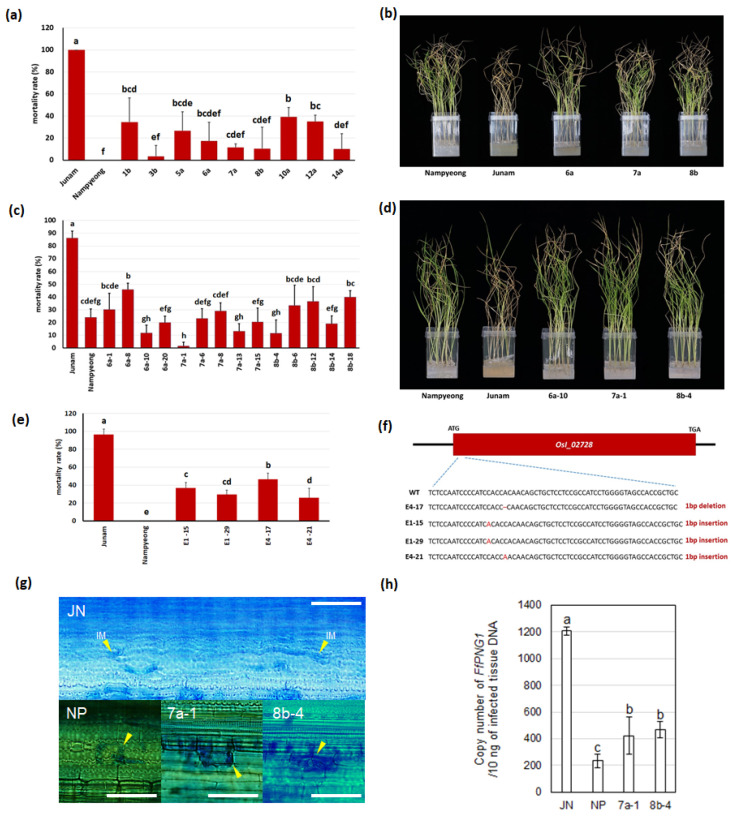
Phenotypes of the *OsI_02728* complementation and gene-edited transgenic lines. (**a**) Mortality rates of the T_0_ complementation transgenic lines after an in vitro bioassay with BD. (**b**) Photograph showing representative lines from (**a**). (**c**) Mortality rates of the T_1_ progeny lines from the plants shown in (**b**). (**d**) Photograph showing representative lines from (**c**). (**e**) Mortality rates of gene-edited lines after an in vitro bioassay with BD. (**f**) Mutations in the gene-edited lines shown in (**e**). WT: wild type. (**g**) Comparison of the invasive mycelial growth of the BD pathogen (*Fusarium fujikuroi* CF283 strain) in Junam (JN), Nampyeong (NP), and two transformants, 7a-1 and 8b-4, harboring *OsI_02728* (*FfR1*). Whereas the fungal pathogen’s vigorous infection and ramification were evident in JN, those in NP, 7a-1, and 8b-4 were extremely limited. Rice stems and sheaths were harvested at 13 dpi. Arrowheads indicate invasive mycelial growth (IM). Scale bars = 50 μm. (**h**) Quantitative evaluation of the propagation of CF283 in the same rice lines retrieved at 13 dpi through Taqman real-time PCR. The copy number of pathogen’s genome in 10 ng of DNA from infected rice tissue was obtained by plotting the crossing points’ values on the standard curve. All analyses were carried out with three replicates. Different letters on the bars indicate statistically significant differences among the lines (Duncan’s multiple range tests; *p* < 0.05).

We examined the genomic region of 93-11 corresponding to the *qFfR1* target region (*1625IND–JNFC13*) of Nipponbare, revealing that it was located at 26.34–26.41 Mb (72.3 kb) in 93-11 (Figure S5). This region harbored seven LRR family genes, including *OsI_02722* and *OsI_02725*, which correspond to *Os01g0601625* and *Os01g0601675*, respectively, which were described above. We performed complementation experiments with the five remaining LRR family genes in this region (*OsI_02726*, *OsI_02728*, *Os2I_02729*, *OsI_02732*, and *OsI_02733*), cloning the genomic regions of these genes from Nampyeong and introducing them into Junam by transformation. While the other transgenic lines showed similar mortality rates to the susceptible Junam variety when infected by the BD pathogen (Figure S1), the nine T_0_ *OsI_02728* transgenic lines showed much lower mortality rates (3.4–39.4%) than Junam (100% mortality) and increased resistance to BD (Figure 2a,b), which indicated that *OsI_02728* is *FfR1*. The T_1_ progeny lines from three T_0_ complementation lines (6a, 7a, 8b) also showed much lower mortality rates (1.7–46.7%) than Junam, which showed an 86.3% mortality rate under the same conditions (Figure 2c,d).

We produced four homozygous knockout mutant lines of *OsI_02728* using gene editing to generate a frame-shift mutation with 1 bp insertions or deletions. These lines showed higher mortality rates than Nampyeong and reduced resistance. The four gene-edited homozygous mutant lines showed mortality rates of 26.0–46.7%, while that of Nampyeong was 0% (Figure 2e,f). This supported the finding that this gene is *FfR1*.

Microscopical observations through aniline blue staining revealed active invasive mycelial growth in Junam. However, this proliferation disappeared in the two transgenic *FfR1* lines (7a-1 and 8b-4) to a similar level to that in Nampyeong. To confirm these results further, we checked the amount of *FfPNG1*, a unigene of *Fusarium fujikuroi*, in the same amount of DNA from infected rice tissue through Taqman real-time PCR. Compared with the fungal growth in the resistant cv. Nampyeong, that in the susceptible Junam was 5.2 times higher. The pathogen’s proliferation in Junam was 2.9 and 2.6 times higher than those in the two transgenic lines (7a-1 and 8b-4) (Figure 2h). These results indicated that the *FfR1* gene inhibits growth of the BD pathogen in rice plants.

### 2.2. The FfR1 Gene and Protein Structures, Subcellular Localization, and Gene Expression Profile

**Figure 3:**
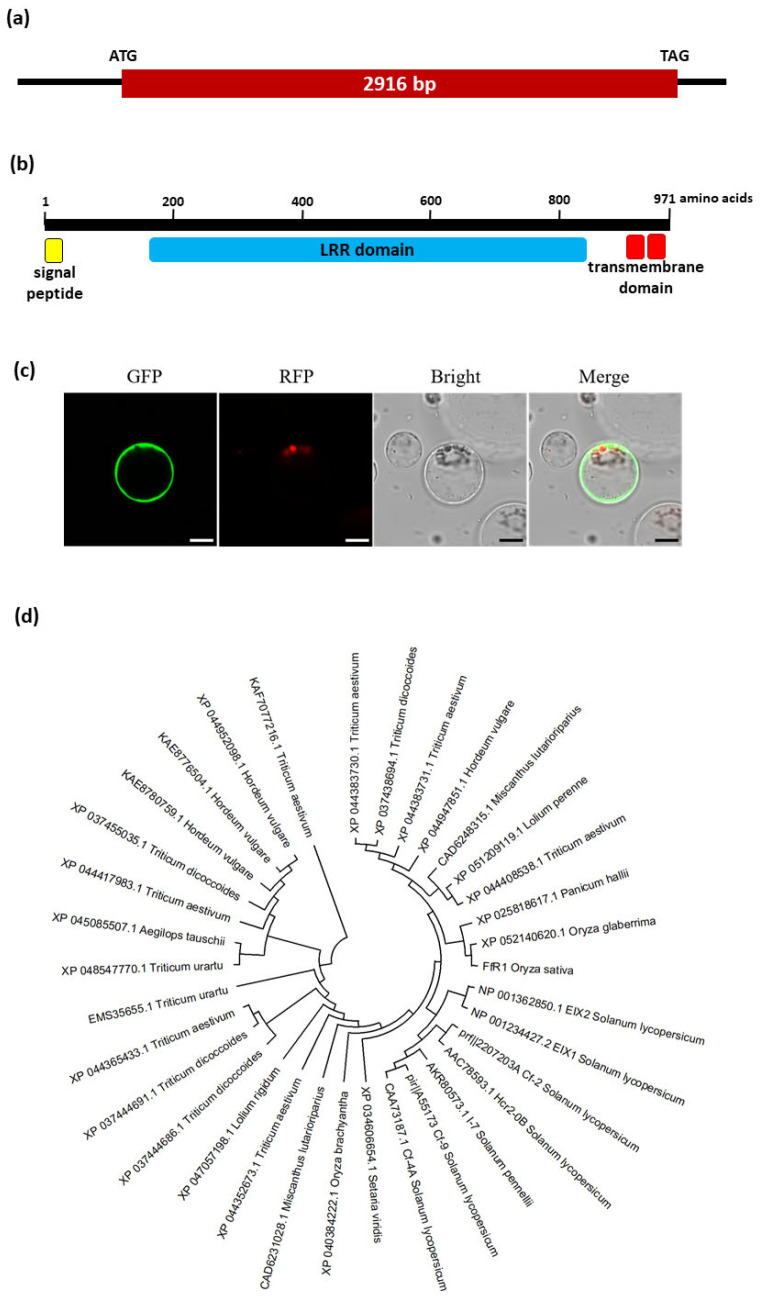
Gene and protein structure of *FfR1* and its subcellular localization. (**a**) Gene structure of *FfR1*. The filled brown box indicates the exon. The start and stop codons are shown at the ends of the exon. (**b**) Protein structure of FfR1. (**c**) Subcellular localization of FfR1. The C-terminal GFP-conjugated FfR1 was expressed in rice protoplasts, which were incubated for 6 h before being imaged under a confocal microscope. RFP signals indicate chlorophyll. Scale bar, 10 µm. (**d**) Phylogenetic relationships of FfR1 homologs. The evolutionary history was inferred using the neighbor-joining method. The bootstrap consensus tree inferred from 1000 replicates was taken to represent the evolutionary history of the proteins analyzed.

The *FfR1* gene is composed of one 2916 bp exon (Figure 3a). The FfR1 protein is composed of 971 amino acids, including an N-terminal signal peptide, an LRR domain of 21 LRR repeats, and two C-terminal transmembrane domains (Figure 3b). This domain structure is similar to that of other LRR receptor-like proteins (LRR-RLPs), such as Cf-2 [37], Cf-4 [38], Cf-5 [39], and Cf-9 [40] from tomato, which have extracellular LRR domains and are anchored in the cell membrane by a transmembrane domain. To observe the subcellular localization of the FfR1 protein, we expressed green fluorescent protein (GFP)-conjugated FfR1 in rice protoplasts and observed the fluorescent signals under a confocal microscope. The signals were mainly observed in the cell membrane (Figure 3c). Phylogenetic analysis was conducted with the proteins that were homologous with FfR1, as identified through BLASTP searches, and known disease-resistant LRR-RLPs. Many homologs of FfR1 were found in various plant species, including *Triticum aestivum* (wheat), *Hordeum vulgare* (barley), *Panicum hallii*, *Miscanthus lutarioriparius*, *Aegilops tauschii*, *Lolium rigidum*, and so on (Figure 3d). Known disease-resistant LRR-RLPs from tomato, such as Cf-2 [37], Cf-4 [38], Cf-9 [40], I-7 [41], LeEix1 and LeEix2 [42], were closely related to FfR1 (Figure 3d).

**Figure 4:**
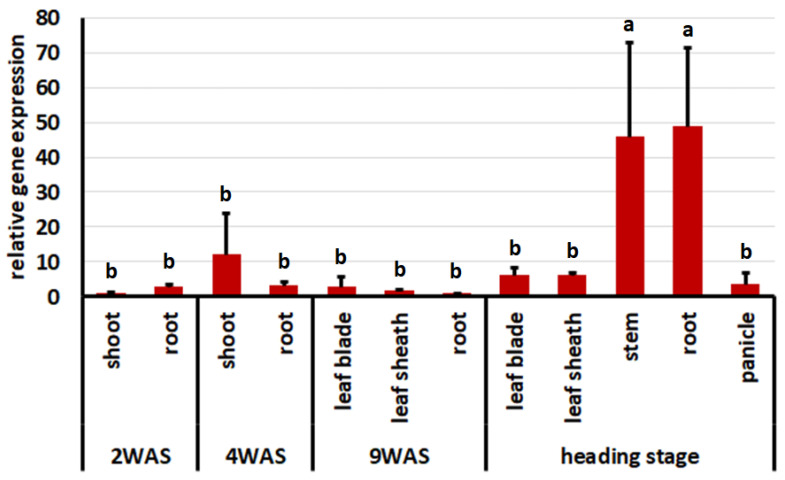
Expression patterns of *FfR1* at different developmental stages and in different organs in Nampyeong. *eEF-1α* was used as an internal control to quantify the relative gene expression levels. Expression levels are expressed relative to the expression level detected in 2-week-old leaves as a baseline. Error bars represent the standard deviation of the expression ratio. 2WAS, 2 weeks after sowing; 4WAS, 4 weeks after sowing; 9WAS, 9 weeks after sowing. Different lowercase letters indicate significant statistical differences at *p* < 0.05.

The expression pattern of the *FfR1* gene at different developmental stages and in different organs in Nampyeong was investigated using qRT-PCR. *FfR1* was expressed in all tested organs and developmental stages. Overall, its expression was highest at the heading stage (Figure 4).

### 2.3. RNA Sequencing Analysis

**Figure 5:**
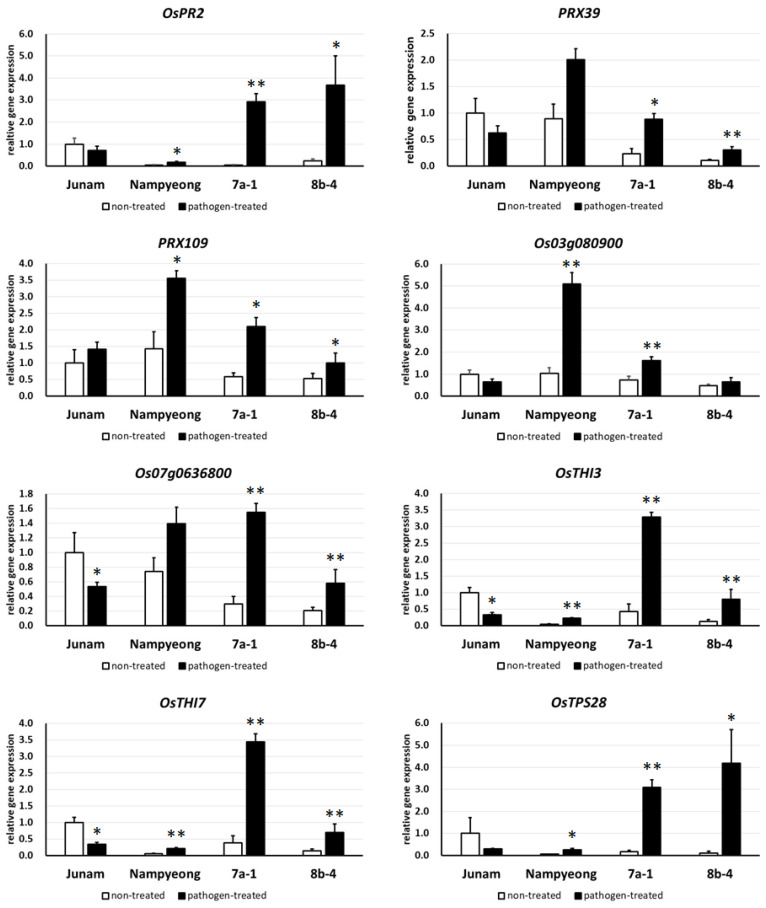
Expression of representative genes showing upregulation in the resistant lines (Nampyeong, 7a-1, and 8b-4) but unchanged or downregulated expression in the susceptible Junam variety. These expression levels were determined using a qRT-PCR analysis, with *eEF-1α* used as the internal control to quantify the relative gene expression levels. Expression levels are expressed relative to the expression levels detected in non-treated Junam as a baseline. Error bars represent the standard deviation of the expression ratio. Asterisks indicate that the expression levels were significantly different between the non-treated and pathogen-treated plants in each variety or line (unpaired *t*-test, * *p* < 0.05 and ** *p* < 0.01).

We performed an RNA sequencing (RNA-seq) analysis with Junam, Nampyeong, and two *OsI_02728* complementation transgenic lines (7a-1 and 8b-4). The statistics of the sequence data are shown in Table S1. For each genotype, 5.4–9.4 Gb of raw sequence data was generated, of which 5.3–9.1 Gb had a quality score higher than Q20. We tested the differential expression of rice genes between non-treated and pathogen-treated samples in both parental varieties and the two complementation transgenic lines using the DESeq2 [43] program (Table S2). To figure out the resistance mechanism of *FfR1*, we extracted the genes that were commonly upregulated in the resistant lines (Nampyeong, 7a-1, and 8b-4) but which were unchanged or had downregulated expression in Junam, with the selection criteria as follows: log_2_ fold change ≤ 0 in Junam, log_2_ fold change ≥ 1, and *p* < 0.05 in Nampyeong, 7a-1, and 8b-4. In total, 57 genes were thus selected (Table S3), which included the pathogenesis-related (PR) genes *OsPR2* and *OsPR1-12*. Two genes encoding dirigent family proteins (*Os03g0809000* and *Os07g0636800*) and two peroxidase family members (*PRX39* and *PRX109*) were also identified, all of which are known to be involved in the biosynthesis of lignin [44,45]. The identified genes also included several that encode thionin proteins (*OsTHI3*, *OsTHI4*, *OsTHI6*, *OsTHI7*, and *OsTHI9*), which are known to be antimicrobial peptides [46], as well as *terpene synthase 28* (*OsTPS28*), which is involved in the biosynthesis of casbene-derived diterpenoids that are implicated in disease resistance in rice [47]. A qRT-PCR analysis was used to validate the expression patterns of 8 of the 57 genes, confirming the accuracy of the RNA-seq data (Figure 5).

**Figure 6:**
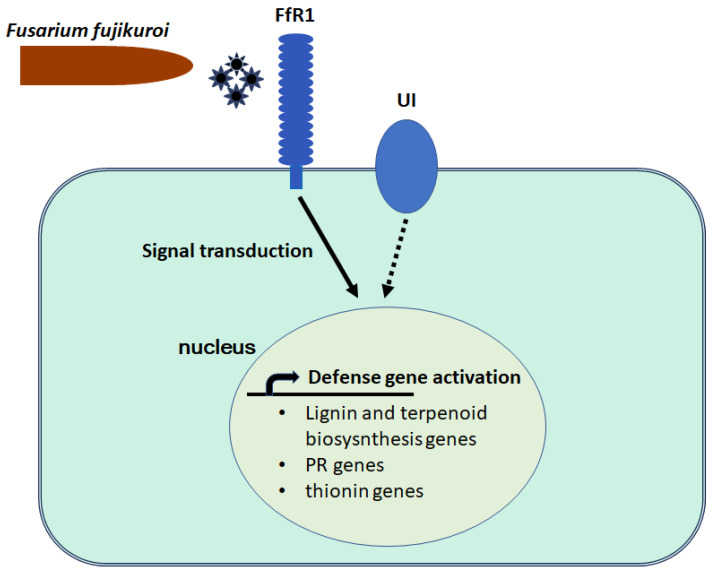
Model of the resistance mechanism driven by FfR1. The FfR1 protein might sense certain molecular patterns from *Fusarium fujikuroi* and subsequently transduce the signal to the nucleus. The defense genes, including lignin and terpenoid biosynthesis genes, PR genes, and thionin genes, are upregulated, which results in the plant’s resistance to the pathogen. UI indicates a protein product of an unidentified resistance gene existing in the Nampyeong variety which should be investigated in a further study.

According to the transcriptomic findings, we can suggest a model to explain the mechanism of resistance driven by FfR1 (Figure 6). The FfR1 protein, located in the cell membrane, might sense certain molecular patterns derived from the BD pathogen *Fusarium fujikuroi* and subsequently transduce the signal to the nucleus. The defense genes, including lignin and terpenoid biosynthesis genes, PR genes, and thionin genes, are upregulated, which results in the plant’s resistance to the pathogen.

### 2.4. Development of the FfR1 Selection Marker

**Figure 7:**
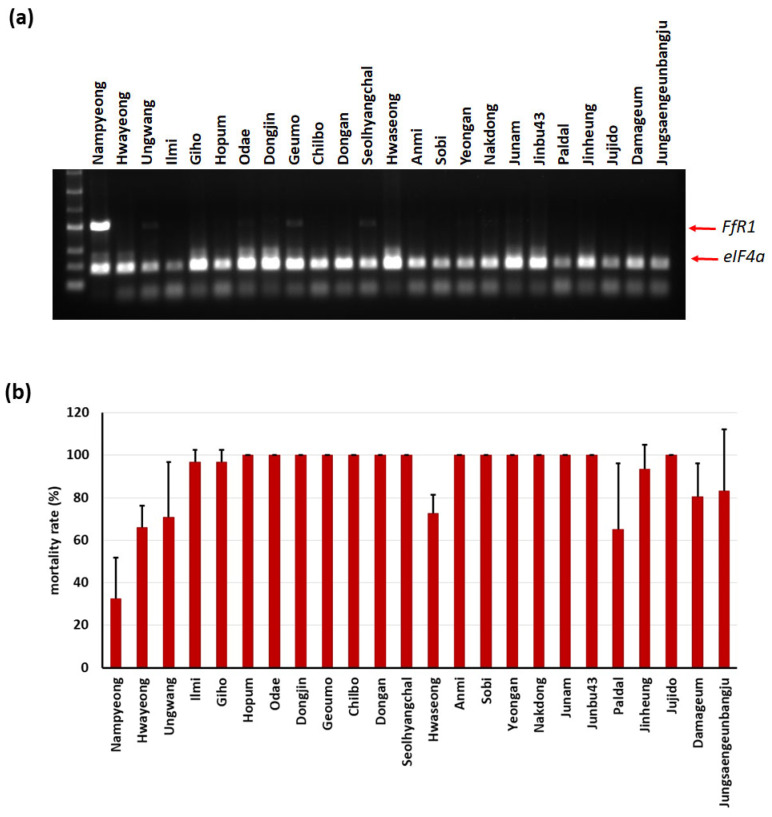
Development of markers for *FfR1*. (**a**) Agarose gel electrophoresis of PCR products generated using the *FfR1* marker in 24 rice varieties. (**b**) Seedling mortality rates of the 24 rice varieties after a BD in vitro bioassay.

We designed an *FfR1* selection marker to enable breeders to target this gene when breeding for BD resistance in rice. Korean rice varieties show presence/absence variation of this gene. We designed a primer pair for this gene (Table S4). In addition, we designed a primer pair for a housekeeping gene, *eIF-4α*, as a PCR control (Table S4). We performed a PCR, placing these two primer pairs in the same well. The varieties with the *FfR1* gene showed two bands: an upper band for *FfR1* and a lower band for *eIF-4α* (Figure 7a). The varieties without the *FfR1* gene showed only the lower band. In an in vitro assay of seedlings with BD, the Nampyeong variety with the *FfR1* gene showed higher resistance, with a lower mortality rate than other varieties without *FfR1* (Figure 7b).

## 3. Discussion

Through map-based cloning, we identified the gene underlying the rice BD resistance QTL *qFfR1*. The *FfR1* gene encodes a member of the LRR-RLP family, many of which play key roles in plant immunity. The LRR-RLP proteins localize to the cell membrane, where they recognize pathogens or pathogen-derived molecules and trigger a signaling cascade that induces the immune response [48]. The tomato *Ve* gene encodes an LRR-RLP protein that confers resistance to *Verticillium* wilt disease caused by *Verticillium dahliae* [49], while the *Cf-2* [37], *Cf-4* [38], *Cf-5* [39], and *Cf-9* [40] LRR-RLP genes in this species enhance plants’ resistance to tomato leaf mold disease, which is caused by *Cladosporium fulvum*. The mechanism can be direct or indirect; for example, Cf-2 senses a pathogen’s attack indirectly by interacting with Rcr3, which is a secreted tomato cysteine protease complexed with Avr2, a cysteine-rich protein secreted by the fungus [50]. The *I-7* [41] gene, also encoding an LRR-RLP, confers the tomato with resistance to *Fusarium* wilt disease caused by *Fusarium oxysporum*, while the tomato (*Lycopersicon esculentum*) *LeEix1* and *LeEix2* LRR-RLP genes are upregulated by the ethylene produced by the plant in response to fungal xylanase, a potent elicitor of plants’ defense responses [42]. In *Arabidopsis thaliana*, the LRR-RLP protein RLP30 was found to perceive the SsE1 elicitor from the pathogen *Sclerotinia sclerotiorum* and is required for resistance against this necrotrophic fungal pathogen [51]. Similar to other LRR-RLPs, we found that FfR1 is also located in the cell membrane (Figure 3c), and future work should examine which aspect of the pathogen or its associated molecules is sensed by FfR1.

LRR-RLPs lacking a cytoplasmic signaling domain interact with SUPPRESSOR OF BIR1-1 (SOBIR1) and BRI1-ASSOCIATED KINASE 1 (BAK1) for their downstream signal transduction [52]. The tomato ortholog of SOBIR1 and its close homolog SOBIR1-like interact in planta with both Cf-4 and Ve1, and are required for the Cf-4- and Ve1-mediated hypersensitive response and subsequent immunity [53], while SERK3/BAK1 also acts as a positive regulator of Ve1 [54]. The findings of the present study suggest that FfR1 induces the transcription of defense genes, including lignin and terpenoid biosynthesis genes, PR genes, and thionin genes (Figure 6); however, the genes involved in the signal transduction pathway from FfR1 to the transcriptional activation of the defense genes are not known. Future studies should elucidate whether rice orthologs of SOBIR1 and BAK1 or other proteins are involved in the signal transduction downstream of FfR1.

The *FfR1* gene was present in Nampyeong, but many other Korean *japonica* rice varieties did not possess this gene. The sequence of the genomic region containing *FfR1* in Nampyeong was identical to that of an *indica* reference variety, 93–11; thus, it was inferred that Nampyeong inherited the *FfR1* gene from an *indica* variety. Within the pedigree of Nampyeong, the Pe-Bi-Hun variety is a Taiwan *indica* variety that is resistant to BD (Figure S6). We tested several other varieties in the pedigree of Nampyeong to determine whether they contain *FfR1* and show resistance to BD, revealing that the *FfR1* gene was transmitted from Pe-Bi-Hun to Nampyeong via Milyang71 and Mangeum, which were resistant to BD. Kanto98 and Chubu2 also showed resistance to rice BD but did not have the *FfR1* gene; therefore, these varieties are likely to possess other resistance genes, which might also have been transmitted to Nampyeong. In the previous QTL analysis, the R^2^ values of *qFfR1* were 0.401 in the DongjinAD/Nampyeong F_2_:F_3_ population and 0.499 in the Junam/Nampyeong F_2_:F_3_ population, providing a further indication that other resistance genes might exist in Nampyeong, despite no other resistance QTLs being found [22,35]. The *FfR1* gene-edited knockout lines derived from Nampyeong did not lose resistance completely, showing mortality rates of 26.0–46.7%, while that of Nampyeong was 0% and that of the susceptible variety Junam was 90% (Figure 2e). It is therefore likely that unidentified resistance genes might exist in Nampyeong in addition to *FfR1*, which should be investigated in further studies.

As to the effect of the environment on BD in rice, the optimum temperature for infection with the pathogen is 27–30 °C and that for development of the disease 35 °C, and wind or water easily carries the conidia from one plant to another [1]. The disease’s incidence was greater in dry nurseries and summer crops than in wet nurseries and spring crops. The expression of BD is favored by high temperatures and elevated CO_2_ levels [1,55]. BD is widespread in tropical as well as temperate environments [1]. BD seems to become more prevalent due to temperature rises caused by climate change in temperate regions. Agronomic measures to control BD include treatment of seed with fungicides, foliar fungicide sprays, treatment of seed with biological control agents such as *Trichoderma* spp., crop rotation, and planting resistant varieties [11]. Recently, *Azadirachta indica* leaf aqueous extract-based silver nanoparticles (AgNPs) displayed inhibition of the mycelial growth of *Fusarium fujikuroi* and potential seed disinfection without toxicity to the seeds during germination, which indicated that AgNPs are an effective alternative strategy for controlling BD [56]. The *FfR1* gene identified through this study will facilitate the development of resistant varieties, which are essential to cope with intensifying BD.

This work is the first to identify a gene underlying a BD resistance QTL in rice. Genetic improvement of the host’s resistance by introgression of resistance genes through the breeding and cultivation of resistant varieties is the most cost-effective and environmentally friendly strategy for controlling plant disease. In this context, the *FfR1* gene can be an effective tool for breeding BD-resistant varieties. Moreover, we have developed a selection marker for the *FfR1* gene based on its sequence. The gene-based marker is better than a QTL-level marker because of the following points. Firstly, its genetic effect will be quite reliable because it was functionally validated by using transgenic approaches, including complementation tests and gene editing. Secondly, because the exact physical location of the gene has been identified, it enables a precision marker-assisted introgression of the target gene without linkage drag caused by the neighboring genes [57]. Therefore, breeders will be able to precisely introduce the *FfR1* gene into other elite rice varieties to develop BD-resistant varieties. Moreover, pyramiding of resistance genes (two or more) in one background is usually used in a breeding program to achieve durable and broad-spectrum resistance [57]. Therefore, it is necessary to identify more BD-resistant QTLs and genes by using map-based cloning or other methods, and develop their gene-based markers, which will be used in pyramiding BD-resistant genes, including *FfR1*.

We found many homologs of FfR1 in various plant species including *Triticum aestivum* (wheat), *Hordeum vulgare* (barley), *Aegilops tauschii*, *Lolium rigidum*, and so on (Figure 3d). In wheat and barley, *Fusarium* head blight (FHB) is a major threat worldwide [58,59]. This disease is also known as scab or head blight, and is caused by *Fusarium graminearum* and other *Fusarium* spp. Although hundreds of QTLs for resistance to FHB have been mapped, identified QTL genes are scarce [60]. The homologs of FfR1 in wheat and barley could be studied as possible candidate FHB-resistant genes. FfR1 shows homology with known LRR-RLPs in tomato such as Cf-2 [37], Cf-4 [38], Cf-9 [40], and I-7 [41], which confer resistance to *Cladosporium fulvum*, which causes leaf mold disease, and *Fusarium oxysporum*, which causes *Fusarium* wilt disease. Therefore, the results of this study could provide clues in research to identify disease resistance genes and reveal their molecular mechanisms in various crops.

In summary, the following points can be studied further on the basis of the results of this study. The associated molecules or molecular patterns of the BD pathogen which are sensed by FfR1 should be elucidated, and yeast two-hybrid (Y2H) screening would be useful for this purpose. The proteins involved in the signal transduction downstream from FfR1 should be elucidated. The rice orthologs of SOBIR1 and BAK1 can be the first candidates for them, and Y2H screening could be used to identify rice proteins interacting with FfR1. It is necessary to identify the unidentified resistance genes other than *FfR1* in the variety Nampyeong. Developing mapping populations by crossing the gene-edited *FfR1* knockout line produced in this study with the susceptible variety Junam and subsequent QTL analysis would enable this work. Identification of more BD-resistant QTLs and genes by using map-based cloning or other methods is also needed, because 30 BD-resistant QTLs have been reported. If the LRR-RLP genes exist in the target QTL region, these could be considered as candidate genes for the QTL. Moreover, pyramiding of BD-resistant genes, including *FfR1*, is needed to develop rice varieties with durable BD resistance.

These results may facilitate new investigations of the molecular mechanisms underlying BD resistance, as well as enabling the breeding of new resistant varieties by providing a BD resistance gene with a precise selection marker, which will contribute to the efficient control of BD which is becoming more prevalent according to temperature rises due to climate change. Further research on host–pathogen interactions, profiling various *Fusarium fujikuroi* strains, investigating patterns of virulence and biochemical and molecular aspects of pathogenesis, and mapping and map-based cloning of new resistance QTLs from various sources of rice germplasm are required.

## 4. Materials and Methods

### 4.1. Plant Materials and Fine-Mapping

The major QTL for BD resistance in rice, *qFfR1*, was discovered in the 22.56–24.10 Mb region of rice chromosome 1 with an F_2_:F_3_ population derived from a cross between a resistant variety (Nampyeong) and a susceptible line (DongjinAD) [35], and was found again in the 21.36–24.37 Mb region on rice chromosome 1 with an F_2_:F_3_ population derived from a cross between Nampyeong and a susceptible variety (Junam) [22]. For fine-mapping of *qFfR1*, we made 12 Junam^*4^/Nampyeong BC_3_F_1_ plants by backcrossing and genotyped them using 103 markers distributed across the whole rice genome. The plant with the highest recurrent recovery rate of the parent genome was selected (Figure S7), and 10 F_2_ progeny plants were grown. These plants were genotyped with four markers showing heterozygous genotypes in the progenitor F_1_ plant. Three plants with the highest genome recovery rates were selected, and their seeds were planted in 200-well growth trays. In total, 2995 Junam^*4^/Nampyeong BC_3_F_3_ seedlings were then transplanted into an experimental field at the National Institute of Agricultural Sciences (Jeonju, Republic of Korea) with 30 × 15 cm spacing between plants.

Genomic DNA was extracted from the plants using a Plant gDNA Extraction Kit (Biomedic, Bucheon, Republic of Korea). All of the plants were genotyped with two markers flanking the target *qFfR1* region, *TQM4* (23.23 Mbp) and *TQM2* (24.10 Mbp), and 153 recombinant plants in the target region were selected. These recombinant plants were genotyped with 12 markers located between *TQM4* and *TQM2*. Among these markers, five markers were developed in our previous studies [22,35], and seven markers were newly developed in this study based on the difference in the genome sequence between Junam and Nampyeong revealed by genome resequencing data analysis. A BD bioassay was also performed using the seeds harvested from the recombinant plants, according to the method used in the previous studies [25,35]. The narrowest interval in which the marker genotypes and the BD response phenotypes co-segregated was chosen as the *qFfR1* gene’s interval. The genome resequencing data from Junam and Nampyeong produced in previous studies were analyzed according to the method described by Ji et al. (2018) [35].

### 4.2. Isolation and Transformation of the Candidate Gene

The genomic region of Nampyeong containing each candidate gene was amplified using the corresponding primer pair (Table S5) using PrimeSTAR GXL polymerase (Takara Bio, Shiga, Japan). The PCR products were purified by gel elution and introduced into a pCAMBIA1300 vector using an In-Fusion HD Cloning Kit (Takara Bio USA, Inc. Mountain View, CA, USA). The transformation vector thus produced was sequence-verified by sequencing the introduced gene, then transformed into the variety Junam, according to a previously reported method [61]. A BD bioassay was performed on the seeds harvested from the transgenic T_0_ plants, according to the method used in previous studies [25,35]. In addition, T_1_ progeny plants from three T_0_ transgenic plants (6a, 7a, 8b) were grown in the greenhouse, and a BD bioassay was performed on the seeds harvested from them.

To knock out the *OsI_02728* gene using genome editing, the guide RNA sequence for CRISPR/Cas9 was designed using Cas-Designer from CRISPR RGEN Tools (http://www.rgenome.net/cas-designer/, accessed on 12 January 2024) [62]. DNA oligos were synthesized to construct guide RNAs with adaptor sequences, and were introduced into the pRGEB32 vector as previously described [63]. The constructed transformation vector was introduced into *Agrobacterium tumefaciens* strain LBA4404, which was used to transform the plants according to a previously reported method [61]. The sequence variations of the *OsI_02728* gene in the gene-edited T_0_ transgenic plants were investigated by Sanger sequencing of the PCR products, including the gRNA target region. Homozygous mutant T_0_ transgenic lines having 1 bp deletions or insertions which caused frame-shift mutations were selected, and the seeds harvested from them were used for a BD bioassay.

### 4.3. Evaluation of the Pathogen’s Ramification in Planta

To evaluate the resistance of rice against BD, we conducted microscopic observations and Taqman real-time PCR. To conduct these experiments, the *Fusarium fujikuroi* strain CF283 was obtained from the Korean Agricultural Culture Collection (KACC; number 46595). The strain was grown on oatmeal agar (50 g oatmeal and 25 g agar per liter) at 23 °C under continuous fluorescent light for 7 days. Macro- and microconidia were harvested with sterilized distilled water. After removal of the mycelial debris via filtering through Miracloth (Millipore, Burlington, MA, USA), the inoculum was adjusted to 2 × 10^5^ conidia mL^−1^ amended with 250 μg mL^−1^ Tween 20. After surface sterilization of rice seeds in a 2% sodium hypochlorite solution for 30 min and washing with sterile distilled water, the seeds were germinated at 28 °C under a 16/8 h fluorescent light regime for a week. The seedlings were further grown hydroponically in a half-strength Yoshida nutrient solution under the same conditions for 3 weeks, and the prepared inoculum was evenly and sufficiently sprayed on the sheaths and stems. The infected seedlings were placed on a plastic platform in transparent, airtight plastic boxes, and the boxes were filled with 100 mL of Yoshida solution. After sealing and tilting the boxes to submerge the roots, the infection progressed for 13 days under absolute humidity. All experiments were performed with 7 replicates. We observed the pathogen’s growth in the rice tissues microscopically through aniline blue staining. The number of fungal genomes in the infected rice stem’s DNA was enumerated via Taqman real-time PCR using a *Fusarium fujikuroi*-specific primer pair (FfPNG1_232F and FfPNG1_355R) and a Taqman probe (Taqman_FfPNG1) in rice tissues retrieved at 13 dpi. Fungal propagation was depicted as the number of *FfPNG1* genes in the 10 ng of DNA of infected rice tissue. Almost all of these experiments were carried out identically to previous works [64].

### 4.4. Subcellular Localization

The full-length cDNA sequence of *FfR1* was inserted into the pDONR221 entry vector, followed by introduction into the pEarleyGate102 vector using the Gateway system (Thermo Fisher Scientific, Waltham, MA, USA), as per the manufacturer’s instructions. The gene was transiently expressed in rice protoplasts, and the subcellular localization of the protein product was determined as previously described [65]. The fluorescence signals were observed using a Leica TCS SP8 confocal laser scanning microscope (Leica Microsystems, Wetzlar, Germany). GFP signals were detected by excitation at 488 nm and emission at 520–560 nm, and RFP signals were detected by excitation at 543 nm and emission at 580–620 nm.

### 4.5. Phylogenetic and Statistical Analysis

The proteins closely related to FfR1 were identified by BLASTP searches (https://blast.ncbi.nlm.nih.gov/Blast.cgi?PROGRAM=blastp&PAGE_TYPE=BlastSearch&LINKLOC=blasthome, accessed on 18 April 2024). Among the proteins homologous to FfR1 thus identified, representative proteins from each plant species were selected and used in phylogenetic analysis with the known LRR-RLPs, including Cf-2, Cf-4, Cf-9, EIX1, EIX2, I-7, and Hcr2-0B. Phylogenetic analysis was conducted by using the MEGA11 program [66].

Statistical analysis for the mortality rates of the transgenic lines was conducted in SAS Enterprise Guide 8.3 (SAS Institute Inc., Cary, NC, USA). Datasets were examined by one-way analysis of variance, and the mean mortality rate of the varieties and transgenic lines were compared using Duncan’s multiple range test at the 0.05 probability level.

### 4.6. RNA-Seq Analysis and qRT-PCR

Seeds from Junam, Nampyeong, and the T_1_ complementation lines (7a-1 and 8b-4) were prepared, sterilized, infected with the CF283 strain of *Fusarium fujikuroi*, and planted on solid Murashige and Skoog medium contained in Incu Tissue jars (SPL Life Sciences, Pocheon, Republic of Korea), as described in previous studies [25,35]. Six Incu Tissue jars were prepared, each containing 20 seeds; three jars held pathogen-treated (infected) plants and three held the non-treated controls (uninfected) plants. After 10 days of infection, the roots of all plants in each jar were collected, and RNA was extracted from the pool. The RNA was extracted using an RNeasy Plant Mini Kit (Qiagen, Hilden, Germany). Thus, six RNA samples were prepared from three pathogen-treated and three non-treated plant root pools from each line and used for the RNA-seq analysis. The total quality and quantity of the RNA were verified using a NanoDrop 1000 spectrometer (Thermo Fisher Scientific, Waltham, MA, USA) and a Bioanalyzer 2100 (Agilent Technologies, Santa Clara, CA, USA). A TruSeq Standard mRNA Sample Preparation Kit (Illumina, San Diego, CA, USA) was used to prepare the sequencing library.

The TruSeq library was sequenced using the HiSeq 2000 platform (Illumina, San Diego, CA, USA), and the RNA-seq reads were mapped to the *Oryza sativa* cv. Nipponbare reference genome (pseudomolecules IRGSP-1.0, https://rapdb.dna.affrc.go.jp/download/irgsp1.html, accessed on 12 January 2024) using HISAT2 [67]. The transcripts’ counts were calculated using featureCounts [68], and a differentially expressed gene (DEG) analysis was conducted using DESeq2 [43]. The RNA sequencing data have been submitted to the Sequence Read Archive (SRA) database of NCBI (https://www.ncbi.nlm.nih.gov/sra, accessed on 27 February 2024) under the accession number PRJNA1073691.

The qRT-PCR analysis for the selected DEGs was performed using the RNA used for the RNA-seq. The cDNA was synthesized using a PrimeScript first-strand cDNA Synthesis Kit (Takara Bio USA, Inc., Mountain View, CA, USA). The primers, shown in Table S6, were designed using the GenScript Real-time PCR Primer Design website (https://www.genscript.com/ssl-bin/app/primer, 21 May 2024). The rice *EUKARYOTIC ELONGATION FACTOR 1α* (*eEF-1α*) gene was used as the reference gene to normalize the transcription levels. PCR amplification was performed in final volumes of 20 μL containing AccuPower 2X GreenStar qPCR MasterMix (BiONEER, Daejeon, Republic of Korea), 10 pmol of each primer, and 2 μL of the cDNA solution, and was run on a CFX96 Real-Time PCR Detection System (Bio-Rad Laboratories, Hercules, CA, USA). Three biological replicates were performed. The relative gene expression levels were calculated using Bio-Rad CFX Manager 3.1 software (Bio-Rad Laboratories).

## 5. Conclusions

We performed fine-mapping of a BD-resistant QTL, *qFfR1*, in the Junam^*4^/Nampyeong BC_3_F_3_ population and delimited its location to the region of the *1625IND–JNFC13* marker (23.63–23.67 Mb; 37.1 kb). Through complementation experiments with seven candidate genes encoding LRR family proteins located in this region, *OsI_02728* was revealed to be the *FfR1* gene and was found to increase resistance to BD. The FfR1 protein has a typical LRR-RLP family protein structure and localizes to the cell membrane. An RNA-seq transcriptome analysis revealed that FfR1 induces the transcription of defense genes, including lignin and terpenoid biosynthesis genes, PR genes, and thionin genes. We developed a selection marker for *FfR1* based on its gene sequence. These results may facilitate the breeding of new BD-resistant varieties by providing a gene for BD resistance with a precise selection marker, which will contribute to the efficient control of BD which is becoming more prevalent according to temperature rises due to climate change.

## Data Availability

Data are contained within the article or Supplementary Materials.

## Supplementary Materials

The following supporting information can be downloaded at: https://www.mdpi.com/article/10.3390/ijms25116214/s1.
