# Supplementary material for: Map-Based Cloning and Characterization of a Major QTL Gene, FfR1, Which Confers Resistance to Rice Bakanae Disease
Source: Int J Mol Sci. 2024 Jun 5;25(11):6214. doi: 10.3390/ijms25116214 (PMC11172731; doi:10.3390/ijms25116214)
Supplement: Supplementary file 1 [file ijms-25-06214-s001.zip › Supplementary_figures.pdf]

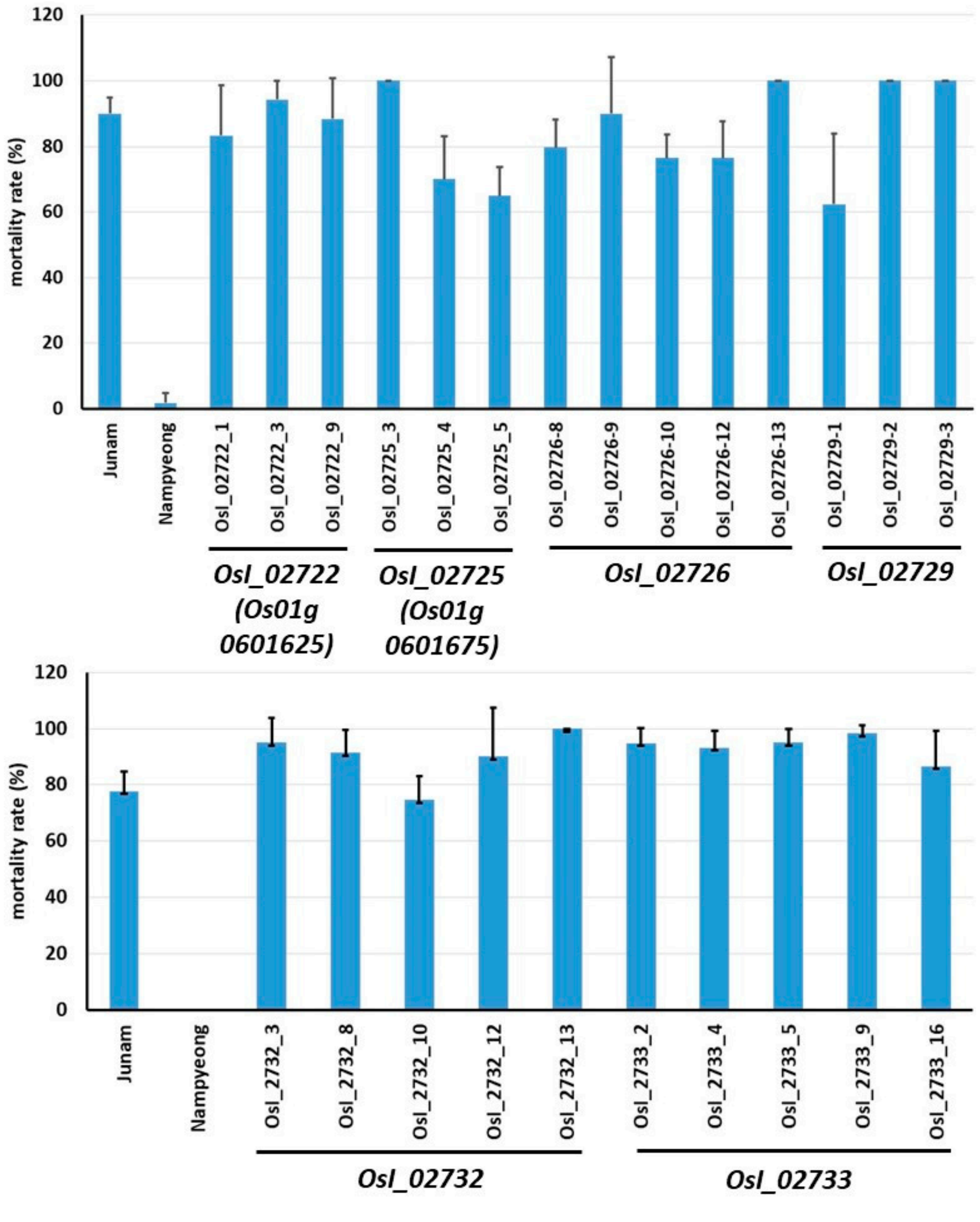

Figure S1. Mortality rates of T0 complementation transgenic lines of candidate LRR genes after bakanae disease in vitro bioassay.

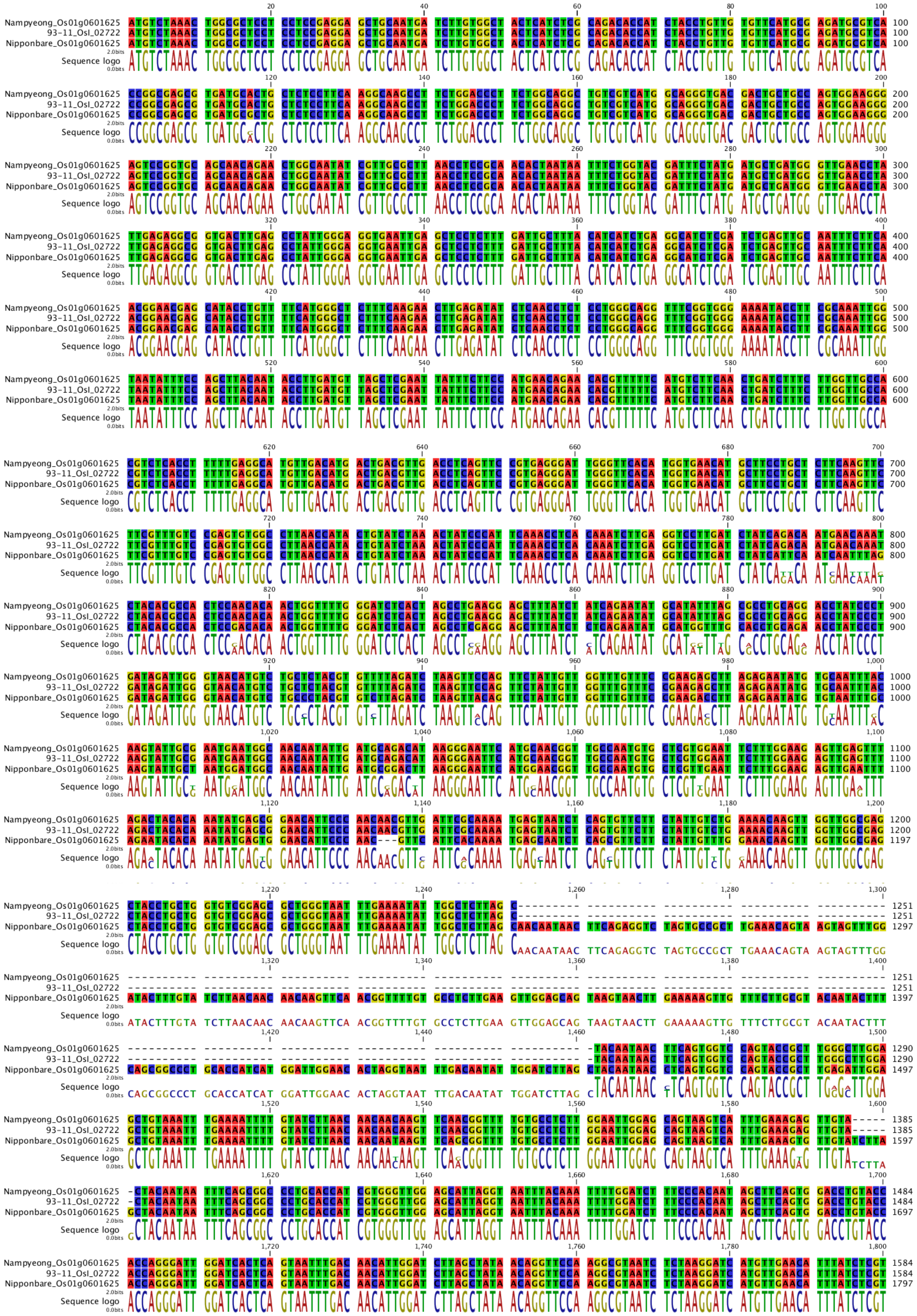
Nampyeong_Os01g0601625
93-11_OsI_02722
Nipponbare_Os01g0601625
Sequence logo

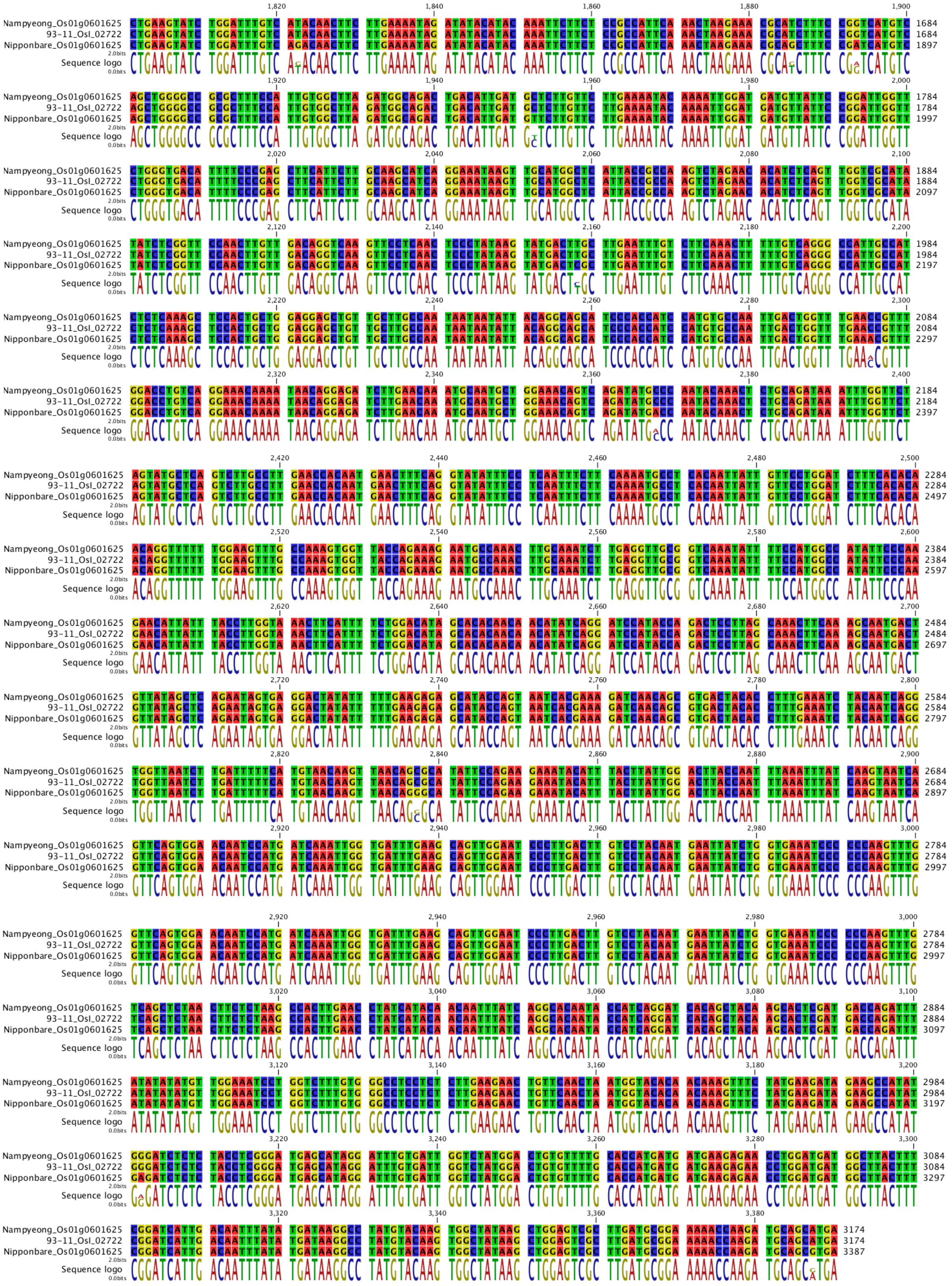

Figure S2. *Os01g0601625* gene sequence alignment among Nippobare, 93-11, and Nampyeong. *OsI_02722* is the gene in 93-11 matching with *Os01g0601625* in Nipponbare.

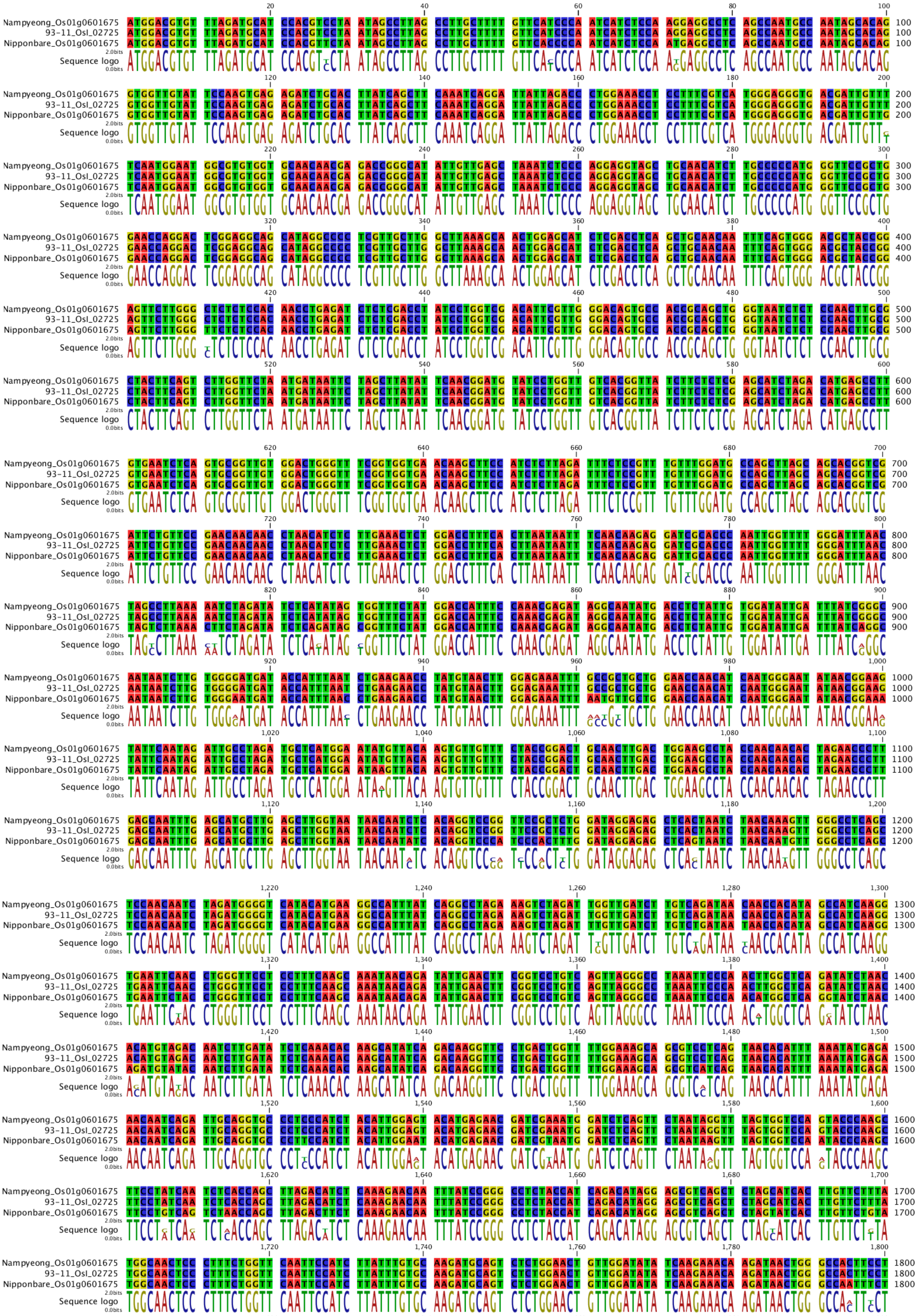
Nampyeong_Os01g0601675
93-11_OsI_02725
Nipponbare_Os01g0601675
Sequence logo

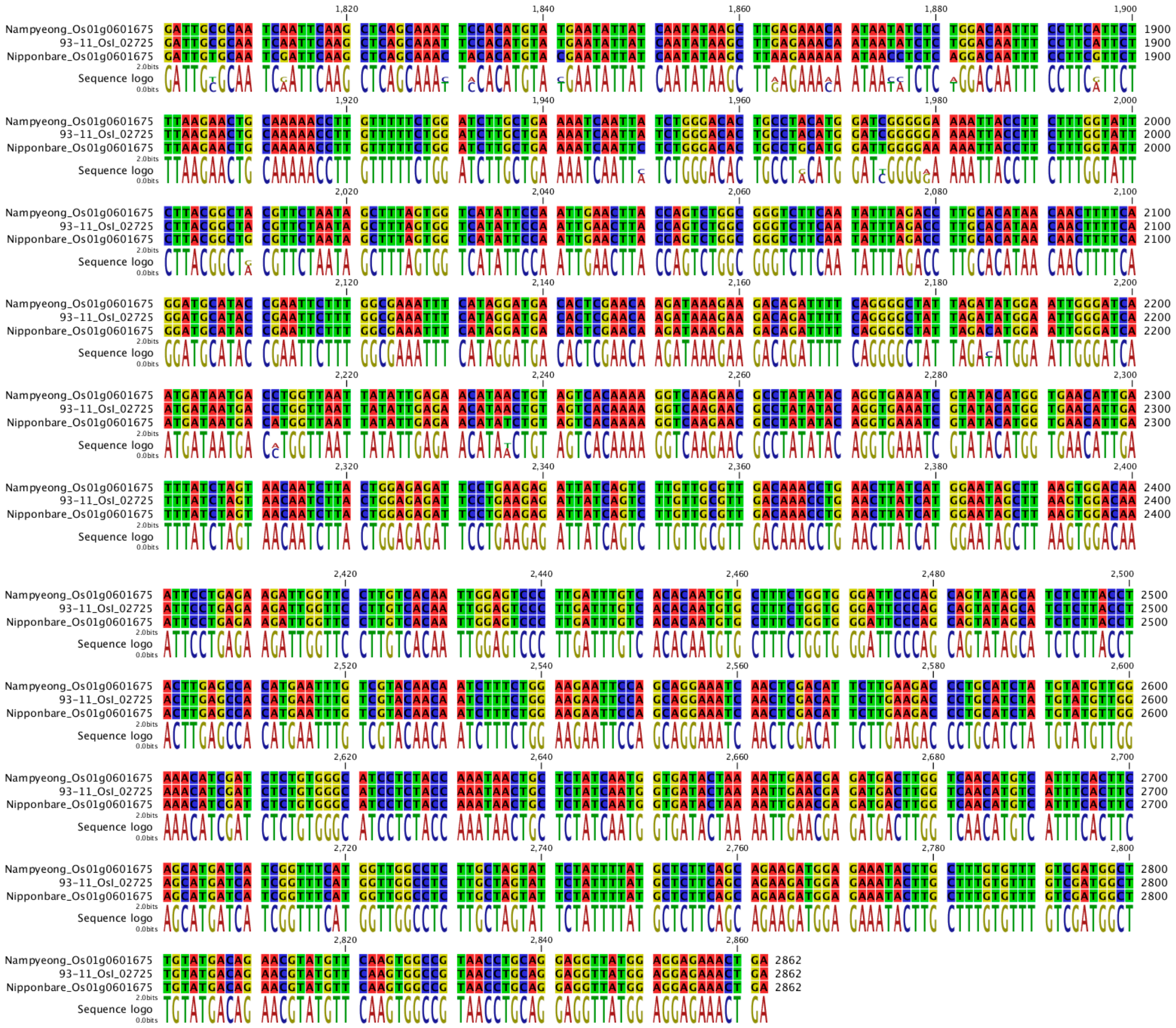

Figure S3. Os01g0601675 gene sequence alignment among Nippobare, 93-11, and Nampyeong. OsI_02725 is the gene in 93-11 matching with Os01g0601675 in Nipponbare.

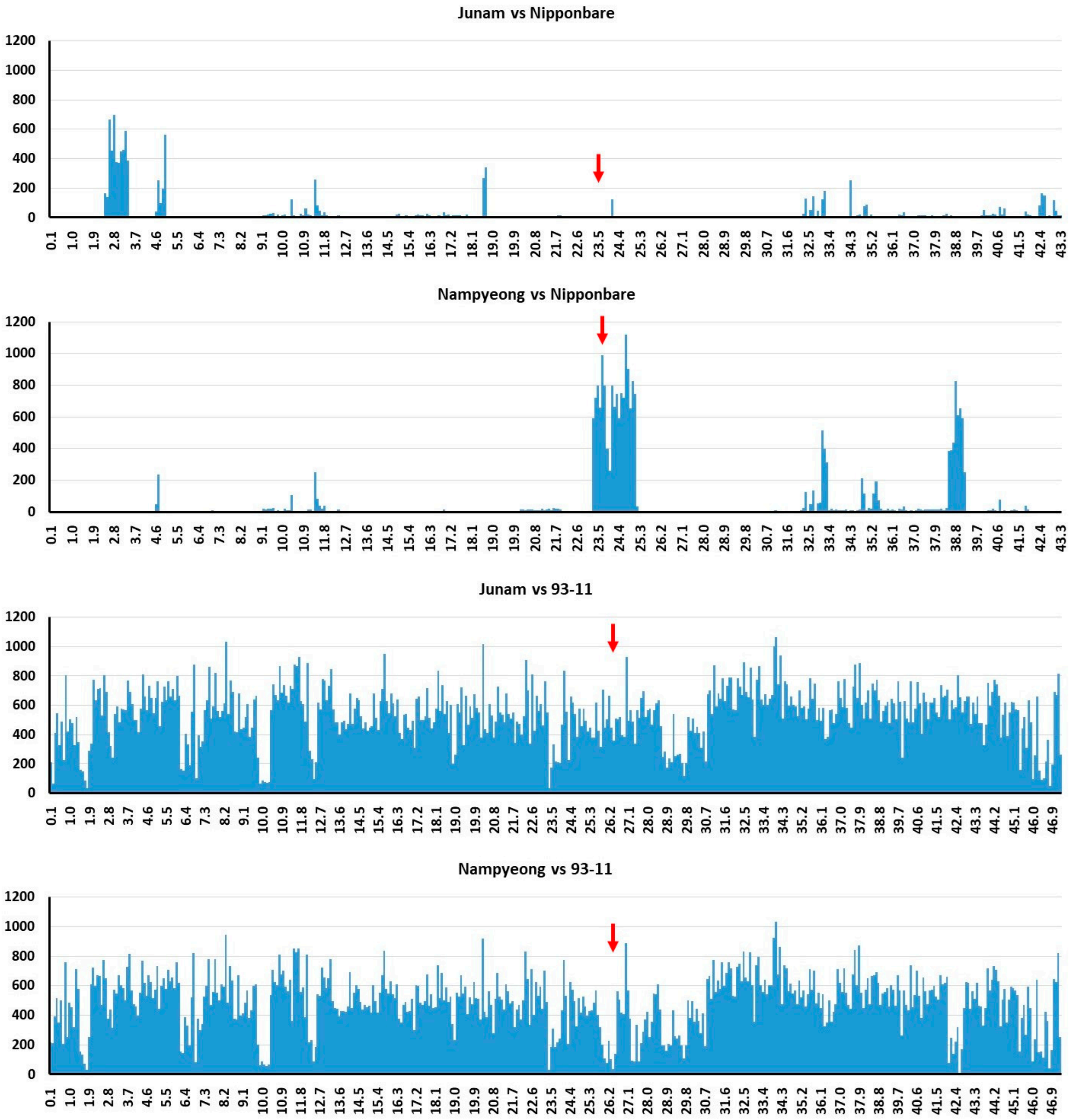

Figure S4. Distribution of SNPs of Junam and Nampyeong in comparison with reference genomes (Nipponbare and 93-11) per 100 kb on rice chromosome 1. The x-axis represents the physical distance along chromosome in mega basepair (Mbp) unit. The y-axis indicates the number of SNPs.

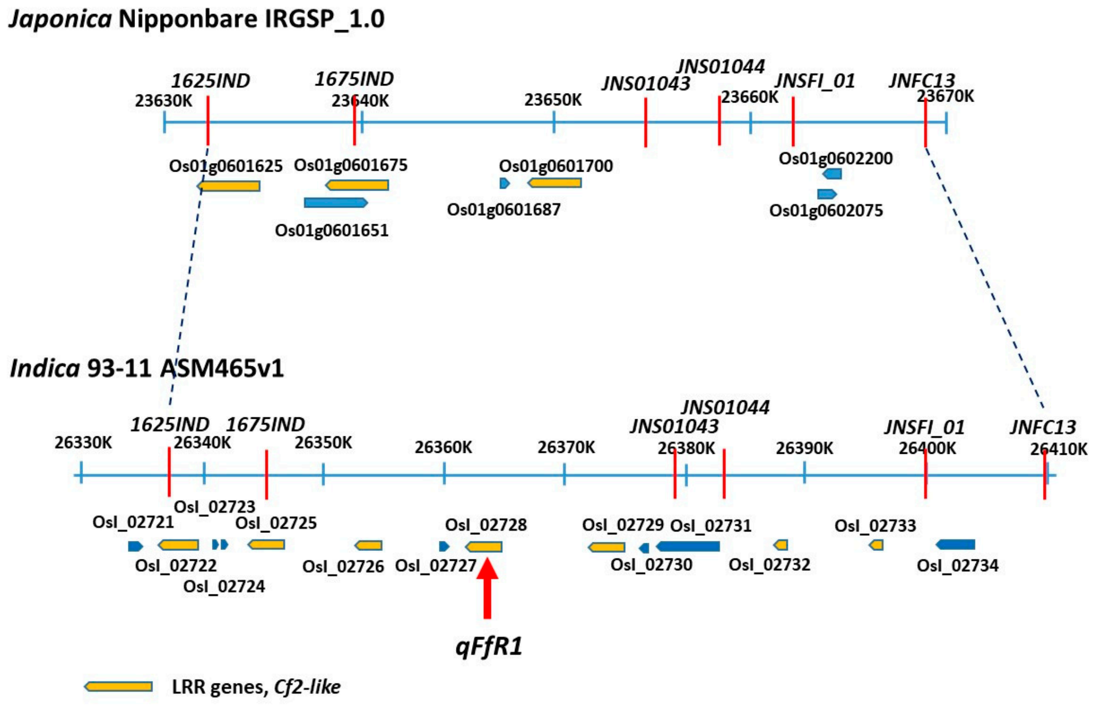

Figure S5. Comparison between *japonica* and *indica* reference genome sequences (Nipponbare vs 93-11) in *qFfR1* genomic region. Red arrow indicate the gene for *qFfR1*.

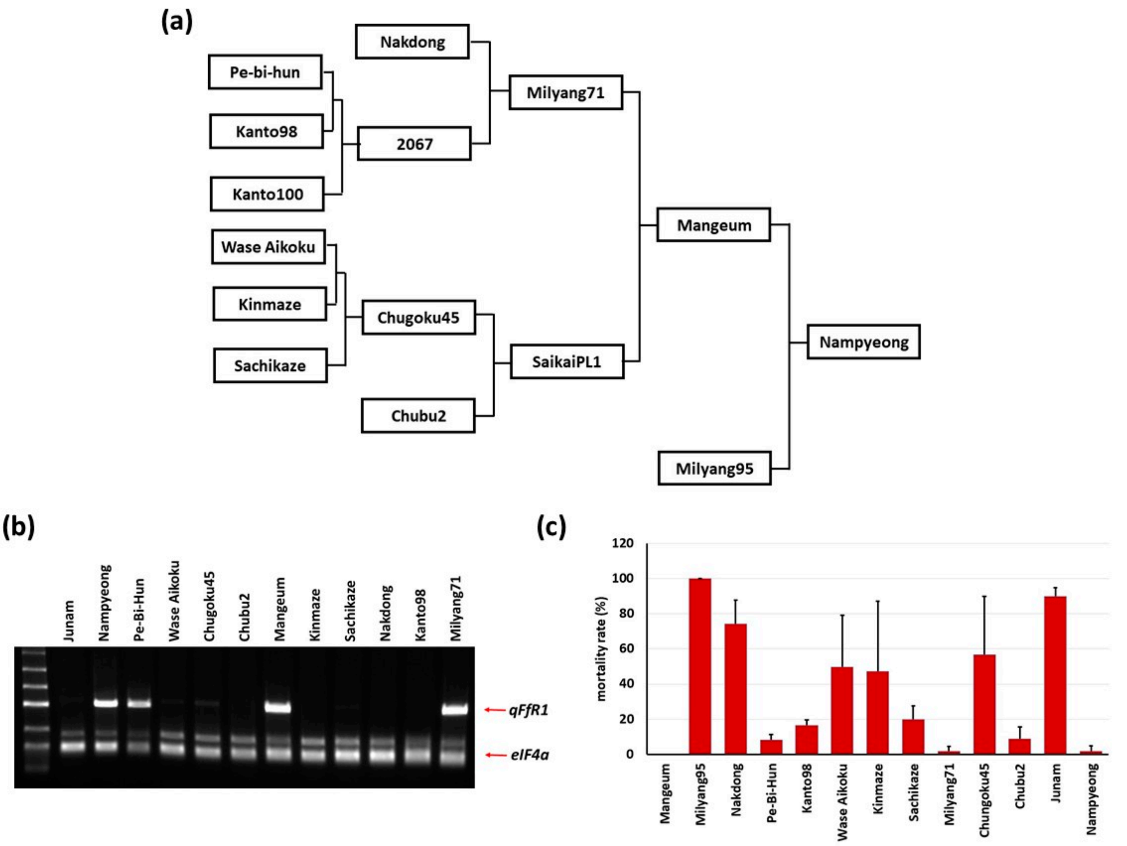

Figure S6. Pedigree of Nampyeong and *qFfR1* gene test. (a) Pedigree of Nampyeong.; (b) *qFfR1* gene test.; (c) Bakanae disease response of ancestor varieties.

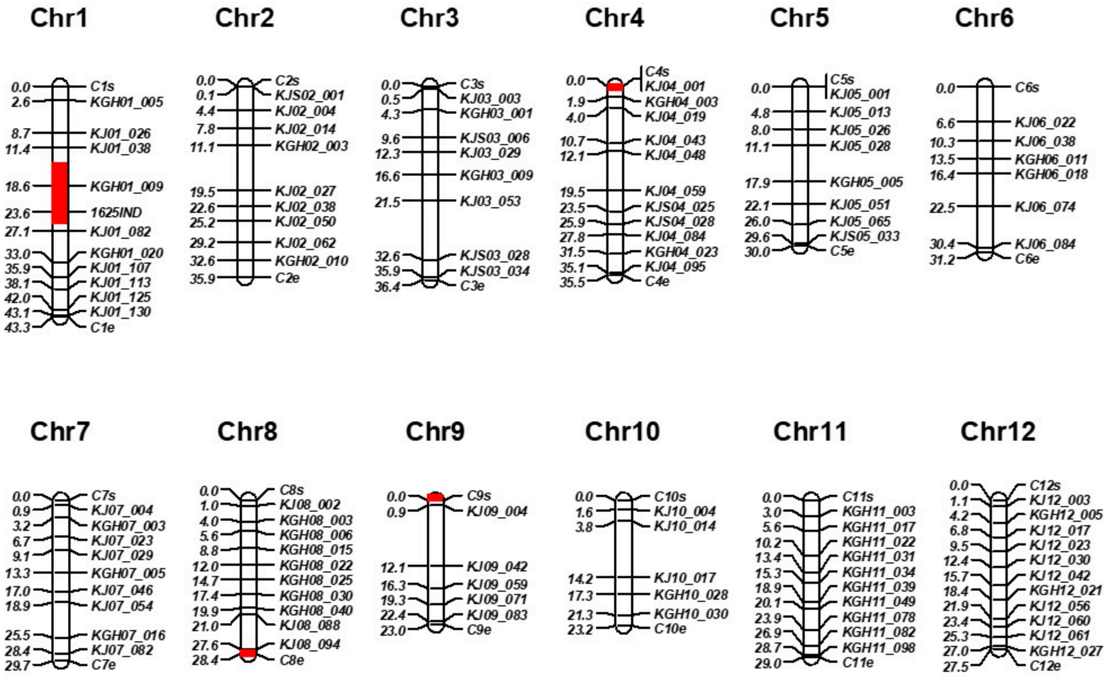

Figure S7. Physical map showing introgression parts of Nampyeong genome in the selected Junam*4/Nampyong BC3F1 plant for fine-mapping population development. 103 KASP markers distributed over whole rice genome were used for genotyping. Red color regions have hetero genotype while white color regions have Junam genotype.
